# Extracellular vesicles from human liver stem cells restore argininosuccinate synthase deficiency

**DOI:** 10.1186/s13287-017-0628-9

**Published:** 2017-07-27

**Authors:** Maria Beatriz Herrera Sanchez, Sara Previdi, Stefania Bruno, Valentina Fonsato, Maria Chiara Deregibus, Sharad Kholia, Sara Petrillo, Emanuela Tolosano, Rossana Critelli, Marco Spada, Renato Romagnoli, Mauro Salizzoni, Ciro Tetta, Giovanni Camussi

**Affiliations:** 1 2i3T, Società per la gestione dell’incubatore di imprese e per il trasferimento tecnologico, Scarl University of Torino, Torino, Italy; 2 Molecular Biotechnology Center, University of Torino, Torino, Italy; 3 Mimetas BV, Leiden, The Netherlands; 4 Department of Molecular Biotechnology and Health Science, University of Torino, Torino, Italy; 5 Molecular and Genetic Epidemiology Unit, Human Genetics Foundation, Torino, Italy; 6 Department of Pediatrics, Regina Margherita Children’s Hospital, University of Torino, Torino, Italy; 7 Liver Transplantation Center, University of Torino, Torino, Italy; 8 Unicyte AG, Oberdorf, NW Switzerland; 9 Department of Medical Sciences, University of Torino, Corso Dogliotti 14, I-10126 Torino, Italy

**Keywords:** Liver transplantation, Citrullinemia type I, Stem cells, Extracellular vesicles

## Abstract

**Background:**

Argininosuccinate synthase (ASS)1 is a urea cycle enzyme that catalyzes the conversion of citrulline and aspartate to argininosuccinate. Mutations in the ASS1 gene cause citrullinemia type I, a rare autosomal recessive disorder characterized by neonatal hyperammonemia, elevated citrulline levels, and early neonatal death. Treatment for this disease is currently restricted to liver transplantation; however, due to limited organ availability, substitute therapies are required. Recently, extracellular vesicles (EVs) have been reported to act as intercellular transporters carrying genetic information responsible for cell reprogramming. In previous studies, we isolated a population of stem cell-like cells known as human liver stem cells (HLSCs) from healthy liver tissue. Moreover, EVs derived from HLSCs were reported to exhibit regenerative effects on the liver parenchyma in models of acute liver injury. The aim of this study was to evaluate whether EVs derived from normal HLSCs restored ASS1 enzymatic activity and urea production in hepatocytes differentiated from HLSCs derived from a patient with type I citrullinemia.

**Methods:**

HLSCs were isolated from the liver of a patient with type I citrullinemia (ASS1-HLSCs) and characterized by fluorescence-activated cell sorting (FACS), immunofluorescence, and DNA sequencing analysis. Furthermore, their differentiation capabilities in vitro were also assessed. Hepatocytes differentiated from ASS1-HLSCs were evaluated by the production of urea and ASS enzymatic activity.

EVs derived from normal HLSCs were purified by differential ultracentrifugation followed by floating density gradient. The EV content was analyzed to identify the presence of ASS1 protein, mRNA, and ASS1 gene. In order to obtain ASS1-depleted EVs, a knockdown of the ASS1 gene in HLSCs was performed followed by EV isolation from these cells.

**Results:**

Treating ASS1-HLSCs with EVs from HLSCs restored both ASS1 activity and urea production mainly through the transfer of ASS1 enzyme and mRNA. In fact, EVs from ASS1-knockdown HLSCs contained low amounts of ASS1 mRNA and protein, and were unable to restore urea production in hepatocytes differentiated from ASS1-HLSCs.

**Conclusions:**

Collectively, these results suggest that EVs derived from normal HLSCs may compensate the loss of ASS1 enzyme activity in hepatocytes differentiated from ASS1-HLSCs.

**Electronic supplementary material:**

The online version of this article (doi:10.1186/s13287-017-0628-9) contains supplementary material, which is available to authorized users.

## Background

Citrullinemia type I is an autosomal recessive disorder caused by a deficiency in argininosuccinate synthase (ASS; common gene symbol alias: ASS1), an enzyme that catalyzes the third reaction of the urea cycle [1]. Typical symptoms that characterize the disorder include hyperammonemia, low plasma arginine levels, and elevated levels of citrulline in the plasma and urine [2, 3]. Furthermore, accumulation of ammonia during the first few days of life leads to poor feeding, vomiting, neuropsychiatric symptoms, and coma [2]. Current treatments involve pharmacologic nitrogen scavenger therapy, life-long protein restriction, and l-arginine supplementation which are aimed towards symptomatic control. For patients that do not respond satisfactorily to these treatments the only alternative is liver transplantation. However, due to the limited availability of donor livers, alternative treatments such as gene therapy are currently under investigation [2].

Previously, we have isolated a population of stem cell-like cells from the human liver known as human liver stem cells (HLSCs) which not only express liver tissue-specific markers, but also mesenchymal and embryonic stem cell markers [4, 5]. Furthermore, under appropriate cell culture conditions, HLSCs demonstrate in vitro differentiation abilities as well as in vivo regenerative properties [4, 5]. For instance, under low gravity culture conditions in the presence of hepatocyte growth factor and fibroblast growth factor 4, HLSCs differentiate into mature hepatocytes [4].

Over the past decade, stem cells have been studied extensively as a form of therapy due to their healing ability. Several preclinical models have been developed out of which a paracrine mechanism of action has been suggested [6, 7]. In the midst of various paracrine mediators identified, extracellular vesicles (EVs) have also been implicated to play a role in the exchange of information [8]. EVs are membrane bound particles secreted by cells in the extracellular milieu. They are classified into two main categories according to their size and subcellular origin. Exosomes are smaller in size (30–100 nm) and are released by exocytosis through the fusion of intracellular multivesicular bodies with the plasma membrane. Microvesicles, on the other hand, are larger (up to 1000 nm) and are shed by direct budding from the cell plasma membrane [8]. Once secreted, these vesicles either remain in the extracellular matrix close to the cell of origin or disperse to other parts of the body through the blood or lymphatic system allowing intercellular exchange of proteins, bioactive lipids, and nucleic acids.

As intercellular vehicles, EVs carry a variety of cargo. For instance, Ratajczak et al. demonstrated that EVs derived from embryonic stem cells may contribute towards the reprogramming of hematopoietic progenitor cells through the delivery of embryonic stem cell-derived proteins and mRNA [9]. Subsequently, other studies followed this up that showed that EVs derived from adult cells also contained and transferred mRNAs and miRNAs to target cells [10, 11].

The ability of EVs to reprogram as a consequence of transferring RNAs has been demonstrated in different tissues and pathologies [12]. For instance, we observed that endothelial progenitor cell-derived EVs activated angiogenic pathways in recipient endothelial cells through horizontal transfer of mRNA [11]. Moreover, in different models of tissue injury, treatments with EVs derived from mesenchymal stem cells have been reported to promote tissue regeneration [13–16]. We also observed that the administration of EVs derived from HLSCs improved the regeneration of liver in rats with a 70% hepatectomy [17] and assisted in the overall recovery of mice in a murine model of acute kidney injury [18].

In this study, we investigated whether EVs derived from normal HLSCs had the ability to carry and transfer wild-type ASS1 to mature hepatocytes differentiated from ASS1-mutated HLSCs, thereby restoring enzyme activity and urea production.

## Methods

### Isolation and culture of normal and mutated human liver stem cells

A liver specimen from a 5-year-old citrullinemia type I patient undergoing liver transplant was obtained under written informed consent from the parents. The tissue was provided anonymously according to the local tissue banking protocol of discarded tissues (# 80911) approved by the University of Torino Ethics Board (Comitato Bioetica di Ateneo).

Hepatocytes were isolated from the liver specimen (5–20 g) by collagenase digestion following the same protocol as for the isolation of normal HLSCs as described previously [5]. Briefly, isolated liver tissues were digested in liver digest medium (Invitrogen, Carlsbad, CA, USA) at 37 °C. The cell suspension obtained was filtered through a sterile 100-μm nylon mesh, centrifuged at 3000 g for 5 min and subsequently washed in cold wash medium (Invitrogen). The hepatocytes obtained were cultured in a medium composed of three parts α-minimum essential medium and one part endothelial cell basal medium-1 (3:1; Lonza, Basel, Switzerland) supplemented with l-glutamine (5 mM), penicillin (50 IU/ml), streptomycin (50 g/ml) (all from Sigma-Aldrich, St. Louis, MO, USA), and 10% fetal calf serum (FCS; Invitrogen). After a few days of culture ASS1-HLSC clones were expanded and subsequently frozen. Wild-type HLSCs were isolated from cryopreserved normal human hepatocytes (hH) obtained from Cambrex Bio Science, Verviers S.p.r.l. (Verviers, Belgium). hH were also used as normal control of mature hepatocytes.

### Isolation of HLSC-derived EVs (HLSC-EVs)

Briefly, 2 × 10^6^ HLSCs and shRNA ASS1 knockdown HLSCs (HLSC shRNA-ASS1) were cultured in serum-free Roswell Park Memorial Institute (RPMI) medium for 18 h at 37 °C. The supernatant was centrifuged for 20 min at 3000 g to remove cell debris and apoptotic bodies. This was followed by a two-step ultracentrifugation protocol whereby the supernatant was firstly centrifuged at 10,000 g (10 K) for 1 h at 4 °C to pellet the 10 K EV fraction and then a second ultracentrifugation at 100,000 g (100 K) for 1 h at 4 °C to isolate the 100 K EV fraction (Beckman Coulter Optima L-90 K, Fullerton, CA, USA) [19]. Both the 10 K and 100 K fractions were resuspended in RPMI supplemented with 1% dimethyl sulfoxide (DMSO) and frozen at –80 °C for later use. The viability of HLSCs at the time of EV collection was 97–99% as determined by trypan blue exclusion.

As suggested by Kowal et al. [20], an iodixanol (Optiprep from Sigma-Aldrich) floating separation protocol was used to further purify the EV fractions from free floating contaminating proteins and RNAs [21]. Briefly, the pellets of EVs obtained after differential ultracentrifugation were directly resuspended in 500 μl 60% iodixanol (Optiprep from Sigma-Aldrich) mixed with 0.25 M sucrose and transferred to ultracentrifuge tubes (10.4-ml polycarbonate centrifuge bottles; Beckman Instruments). Subsequent gradients of iodixanol at 30%, 15%, and 5% were then layered on top of the initial 60% EV-iodixanol/sucrose preparation and the final volume adjusted to 10 ml by topping up with saline solution. The tubes were then ultracentrifuged at 350,000 g for 1 h, 4 °C, without braking in an Optima L-100 K ultracentrifuge (Beckman Coulter) equipped with type 90Ti rotor. Following ultracentrifugation, the 15%, 30%, and 60% fractions were recovered, diluted with phosphate-buffered saline (PBS), and ultracentrifuged again at 100,000 g for 1 h. The pellet obtained was resuspended in RPMI supplemented with 1% DMSO and either used fresh or stored for subsequent studies under –80 °C conditions. No difference in biological activity was observed between fresh and stored EVs.

In order to study the internalization of EVs by fluorescent microscopy, EVs were labeled with 1 μM Dil dye (Thermo Fisher Scientific, Waltham, MA,USA) as described previously [18]. Briefly, purified EVs were resuspended in PBS supplemented with 1 μM Dil dye and ultracentrifuged at 100,000 g for 1 h at 4 °C. Following labeling, the EVs were washed once more with PBS by ultracentrifugation as mentioned above. The pellet obtained was then resuspended in RPMI with 1% DMSO and frozen for subsequent studies.

Concentration and size distribution of EVs were determined by the Nanosight LM10 system (NanoSight, Wiltshire, UK). Briefly, EV preparations were diluted (1:200) in sterile saline solution and analyzed by the Nanoparticle Analysis System using the NTA 1.4 Analytical Software [21].

### Flow cytometric analysis

Cytofluorometric analysis was performed as described previously [4]. The antibodies used (conjugated with phycoerythrin (PE) or fluorescein isothiocyanate (FITC)) are as follows: anti-CD45, anti-Albumin, anti-CD90, anti-CD73, anti-CD105, anti-CD29, anti-CD31, anti-KDR, and anti-VE-Cadherin (Dako Denmark A/S, Copenhagen, Denmark). All incubations with antibodies were performed in 100 μl PBS containing 0.1% bovine serum albumin (BSA) at 4 °C. The cells were washed twice between incubations and analyzed (10,000 cells/sample) on a BD FACSCalibur cytometer (BD Biosciences Pharmingen, San Jose, CA, USA). For detection of albumin, cells were permeabilized and labeled with anti-albumin monoclonal antibody (R&D Systems, Abington, UK) followed by PE-conjugated anti-mouse IgG secondary antibody. All samples were gated on the basis of negative controls, and compensated appropriately prior to analyses. Population percentages and numbers were generated for gated populations from each experiment using Cell Quest software (BD Biosciences Pharmingen).

### In vitro osteogenic, adipogenic, and endothelial differentiation

Osteogenic, adipogenic, and endothelial differentiation of ASS1-HLSCs was performed as described previously [4]. Briefly, ASS1-HLSCs were cultured in osteogenesis induction medium (Lonza) for 3 weeks (replenished twice per week for 3 weeks). Differentiation was evaluated by fixing cells with 4% paraformaldehyde for 20 min and then staining with alizarin red, pH 4.1 (Sigma-Aldrich), at room temperature. For adipogenic differentiation, cells were cultured in adipogenesis induction medium (Lonza), followed by adipogenic maintenance medium (Lonza). To evaluate the differentiation, cells were fixed with 4% paraformaldehyde for 20 min at room temperature then stained with 0.5% oil red O (Sigma-Aldrich) in methanol (Sigma-Aldrich). Endothelial cell differentiation was obtained by culturing ASS1-HLSCs in EBM (Lonza) with vascular endothelial growth factor (10 ng/ml; Sigma-Aldrich) for 10 days. Following culture, endothelial differentiation was evaluated by flow cytometric analyses of endothelial markers as described previously [4].

### Immunofluorescence analysis

Indirect immunofluorescence of cells was performed as described previously [4]. Briefly, ASS1-HLSCs cultured on chamber slides (Nalge Nunc International, Rochester, NY, USA) were fixed with 4% paraformaldehyde and/or permeabilized (only for intracellular markers) with 0.1% Triton X-100 buffer. Cells were then labeled with the following monoclonal antibodies: anti-albumin (R&D Systems), anti-nestin (BD Biosciences Pharmingen), anti-α-fetoprotein (αFP; R&D Systems), anti-nanog (Abcam, Cambridge, MA), anti-Sox2 (Abcam), anti-vimentin (Sigma-Aldrich), anti-cytokeratin 8 (CK8), anti-CK19, anti-Von Willebrand factor (all from Sigma-Aldrich), and anti-ASS1 (Thermo Fisher Scientific). Alexa Fluor 488 anti-rabbit IgG and Texas Red anti-mouse IgG (Thermo Fisher Scientific) were used as secondary antibodies and Hoechst 33258 dye (Sigma-Aldrich) was applied for nuclear staining. Labeling of cells with only secondary antibodies or substitution with nonimmune rabbit, rat, or mouse IgGs served as controls. Slides were analyzed by confocal microscopy using a Zeiss LSM 5 Pascal Model Confocal Microscope (Carl Zeiss International, Jena, Germany).

### In vitro culture in RCCS and urea production

In order to differentiate HLSCs and ASS1-HLSCs into functional hepatocytes, cells were cultured in microgravity conditions using the Rotary Cell Culture System (RCCS; Synthecon Incorporated, Houston, TX, USA) as described previously [4]. Briefly, cells (250,000/ml) were resuspended in medium consisting of 75% Dulbecco’s modified Eagle’s medium (DMEM; Mediatech, Herndon, VA, USA), and 25% MCDB 201 (Sigma-Aldrich) supplemented with 2% fetal bovine serum (FBS), linoleic acid (Sigma-Aldrich), l-ascorbic acid (Sigma-Aldrich), insulin-transferrin-selenium (ITS; Sigma-Aldrich), penicillin (100 μg/ml), streptomycin (100 μg/ml), and 10 ng/ml of hepatocyte growth factor and fibroblast growth factor 4; 10 ml of the cell suspension was then loaded into circular RCCS vessels and rotated about their own axis at 8–10 rotations per minute under 37 °C conditions. After 4 days of culture, the supernatant was collected, centrifuged at 300 g for 5 min and stored at –20 °C. Levels of urea in the supernatants were then evaluated using the blood urea nitrogen (BUN) colorimetric detection kit as per the manufacturer’s protocol (Arbor assays, MI, USA). For selective RCCS experiments, cells were cultured in the presence of HLSC-EVs with or without α-methyl-dl-aspartic acid (MDLA), a specific inhibitor of ASS1 enzyme. Treatment of ASS1-HLSCs with ASS1 shRNA EVs or fibroblast EVs (hFib EV) served as controls.

### Western blot analysis

The level of ASS1 enzyme in cells was determined by Western blot analyses. Briefly, cells were lysed on ice for 30 min in radioimmunoprecipitation assay (RIPA) buffer (50 mM Tris-HCl PH 7.4, 150 mM NaCl, 1% NP-40, 0.1% SDS; Sigma-Aldrich) supplemented with the protease inhibitors phenylmethylsulfonyl fluoride (PMSF) (1 mM), leupeptin (10 μg/ml), and aprotinin (100 units/ml). Proteins from EVs were isolated using the all-in-one purification kit (Norgen Biotek Corp, TO, Canada) according to the manufacturer’s protocol, and the concentrations determined by the Bradford method.

Protein samples at a concentration of 10–30 μg were separated in 8% or 4–15% gradient SDS-PAGE gels under reducing conditions and electroblotted onto 0.2-mm nitrocellulose membranes (GE Healthcare Life Sciences, MA, USA). The membranes were blocked in Tris-buffered saline-Tween (TBS-T; 25 mM Tris, pH 8.0, 150 mM NaCl, and 0.05% Tween-20) containing 5% (w/v) nonfat dried milk for 1 h. After blocking, membranes were probed overnight with mouse anti-ASS1 (BD Transduction Laboratories), mouse anti-CD63, rabbit anti-tubulin, and goat anti-actin (Santa Cruz Biotechnology, CA, USA). After extensive washings with TBS-T, the blots were incubated with appropriate peroxidase conjugated secondary antibodies (goat anti-mouse, goat anti-rabbit, and mouse anti-goat IgG; Pierce, Thermo Fisher Scientific) for 1 h at room temperature. Following incubation, the membranes were washed extensively with TBS-T, probed with enhanced Super Signal West Femto Maximum Sensitivity Substrate (Thermo Fisher Scientific), and detected by the Chemidoc system (Bio-rad, CA, USA).

### Immunoprecipitation and enzymatic activity of ASS1

Cells were lysed in nondenaturing lysis buffer (137 mM NaCl, 2 mM EDTA, 20 mM Tris–HCl, pH 7.5, 10% glycerol, 1% Nonidet P-40, and protease inhibitor cocktail) and centrifuged at 300 g for 15 min at 4 °C. The supernatants were collected and quantified for total protein concentration using the Bio-Rad protein assay method.

Immunoprecipitation of ASS1 was performed using Dynabeads Protein G (Thermo Fisher Scientific). Briefly, the beads were incubated with 10 μg ASS1-antibody (Thermo Fisher Scientific) per mg of total protein for 1.5 h at 4 °C to form complexes. The bead/ASS1-antibody complexes were washed with PBS-0.02% tween and incubated with cell lysates (1 mg total protein) overnight at 4 °C under rotation. After three washes with PBS-0.02% tween, ASS1 proteins were eluted by adding 20 μl 50 mM glycine at pH 2.8. In order to prevent the loss of the tridimensional structure of ASS1, 200 μl TRIS-HCL 20 mM pH 7.8 was added.

Eluted proteins were then evaluated for ASS1 activity using a modified version of the assay described by Lakhal-Naouar et al. [22]. Briefly, eluted protein samples (12.5 μl per reaction) were resuspended in reaction buffer (20 mM Tris-HCl, pH 7.8, 4 mM ATP, 4 mM citrulline, 4 mM aspartate, 6 mM MgCl2, 20 mM KCl, and 0.2 units of pyrophosphatase) to a final volume of 20 μl. The reaction samples were then transferred to 96-well microtiter plates in duplicates and incubated for 30 min at 37 °C. Following incubation the reaction was stopped by adding 20 μl malachite green reagent (Malachite Green Phosphate Assay Kit, Bioassay System, CA, USA). Reactions without citrulline and aspartate substrates were prepared to serve as controls. Phosphate accumulation was determined at 655 nm by spectrophotometry, and the concentration interpolated from a standard curve of inorganic phosphate (Pi). Due to the spontaneous release of Pi in the absence of substrates, the final concentration of Pi from affinity-purified proteins and/or rat liver extract (RLE; served as positive control for the reaction) were determined by subtracting the mean concentration of Pi obtained in the absence of substrates from the mean concentration of Pi obtained in the presence of substrates. The specific ASS1 activity was determined using the following formula: nanomoles of phosphates released/mg protein/30 min [22]. In selected experiments, ASS1-siRNA transfected ASS1-HLSC were used.

### RNA extraction and qRT-PCR

Total RNA from cells was extracted using TRIzol reagent (Thermo Fisher Scientific) and total RNA from HLSC-EVs was isolated using the all-in-one purification kit (Norgen Biotek Corp.) according to the manufacturer’s protocol. The quantity and quality of isolated RNAs were determined using the Nanodrop ND-1000 spectrophotometer (Thermo Fisher Scientific). cDNA was then synthesized using the High Capacity cDNA Reverse Transcription Kit (Thermo Fisher Scientific) as per the manufacturer’s protocol. cDNA at a concentration of 2 ng was used per sample per quantitative real-time polymerase chain reaction (qRT-PCR) reaction in triplicate using a 96-well StepOne Real-Time System (Thermo Fisher Scientific). Wells containing only primers were cycled in parallel with each run and served as negative cDNA controls. For mRNA expression in cells, results were analyzed using the RQ method with GUSB gene as an internal control and for HLSC-EVs, the 2^–∆Ct^ method was adopted using S18 gene as internal control. Primers and probes were designed using the Universal ProbeLibrary Assay Design Center software (www. lifescience.roche.com). Primers used to amplify ASS1 mRNAs are summarized in Table 1.

**Table 1:** Primers used in qRT-PCR to evaluate ASS1 mRNA expression

PCR primers	Primer sequences (5′–3′)
hASS1-Iso1	**F1** TGT GAA AAC AGA TTC CACG C
	**R1** CCA ATG TTG GCC AGA TAG GC
hASS1-Iso2	**F1** ACG CTA TGT CCA GCA AAG GC
	**R1** CCA ATG TTG GCC AGA TAG GC
hASS1-Exon 14 (Primer 1)	**F** AAGTCCCAGGAGCGAGTGG
	**R** GTGGGGCACCTACCTCACC
hASS1-Exon 15 (Primer 2)	**F** GCCAGGCTGAGCTGACAAG
	**R** ACCTGGAGGCTCTGAAGGC

*ASS* argininosuccinate synthase, *F* forward, *qRT-PCR* quantitative real-time polymerase chain reaction, *R* reverse

### ASS1 silencing

A set of five pLKO.1 HIV-based lentiviral vectors targeting the human ASS1 gene were purchased (TRCN0000045553, TRCN0000045554, TRCN0000045555, TRCN0000045556, TRCN0000045557; Dharmacon RNAi Consortium (TRC) Lentiviral shRNA). shRNAs were tested on HLSCs to evaluate the silencing efficiency and the most efficient and stable shRNA was used to specifically downregulate ASS1 expression in HLSCs. The lentiviruses pLKO.1-scr (expressing a “scramble” shRNA as control) and pLKO.1-ASS1 (expressing the shRNA specific for ASS1) were produced in HEK293FT cells using a lentiviral packaging system after which HLSCs were infected with the lentiviruses in the presence of Sequa-brene (Sigma-Aldrich). Following infection, cells were selected with 5 μg/ml puromycin. The efficacy and stability of silencing in selected cells was evaluated by qRT-PCR.

### siRNA transfection

ASS-siRNA and nonsilencing siRNA were purchased from Thermo Fisher Scientific. Transient transfection of ASS-siRNA was performed using Lipofectamine RNAiMAX transfection reagent (Invitrogen) as per the manufacturer’s protocol. ASS1-HLSCs (500,000 cells per well) were seeded in a petri dish (10-cm diameter) and cultured for 24 h. The complete culture medium was replaced with antibiotic-free medium and transfected with 300 pM ASS-siRNA or nonsilencing RNA for 3 days in the presence of Lipofectamine RNAiMAX. Before the addition in cell cultures, siRNAs were incubated with lipofectamine for 20 min at room temperature in Opti-Mem medium (Invitrogen). Preliminary experiments using FITC-siRNA (Qiagen, Valencia, CA, USA) indicated that the peak of expression was 3 days after transfection. After 3 days of transfection, ASS1-HLSCs were detached with trypsin and 3.0 × 10^6^ cells were cultured in RCCSs in the presence of 1 × 10^10^ EV-HLSCs. The effective ASS-siRNA transfection was evaluated in ASS1-HLSCs by RT-PCR. ASS enzymatic activity was measured after 4 days of culture under RCCS culture conditions.

### DNA mutation analysis

Genomic DNA was extracted using DNeasy Blood & Tissue Kit (Qiagen) according to the manufacturer’s instructions. The quality and quantity of extracted DNA was determined using the Nanodrop ND-1000 spectrophotometer (NanoDrop Technology).

Two regions of the human ASS1 gene located in exons 14 and 15 were amplified by PCR. These regions comprise of the following codon mutations: R363W (exon 14) and G390R (exon 15). The primers were developed (Table 2) based on the GenBank reference sequence (accession no. NC_000009.11), using Primer Express 3.0 software (Thermo Fisher Scientific). PCR was then performed in a final volume of 50 μl, containing 10× PCR buffer, 0.05 units/μl JumpStart Taq polymerase, 10 mM deoxynucleotide triphosphates mix, 150 ng genomic DNA, and 500 nM primers. Cycling conditions were as follows: 1 min at 94 °C, 35 cycles at: 94 °C for 30 s, 62 °C for 30 s, and 72 °C for 60 s, followed by 1 min at 72 °C. At the end of the reaction, 10 μl PCR-amplified DNA samples were analyzed using 1% agarose gel.

**Table 2:** SNaPshot primers

SNaPshot primers	SNaPshot primer sequences
R363W	T_20_GGTGTACATCCTCGGC
G390R	T_30_GCCAACTGATGCCACC

R363W was used to amplify the genomic region containing the codon mutation g.55277 C > T and primer G390R for the region containing the mutation g.59839 G > A

To evaluate the presence of DNA in EVs derived from HLSCs, DNA was extracted and analyzed by PCR. Briefly, 14 different preparations of EVs obtained from HLSCs were pooled together by ultracentrifugation at 100,000 g for 2 h at 4 °C. The supernatant was discarded and lysis buffer was added directly to the EV pellet and processed as per the manufacturer’s protocol to isolate the DNA (DNeasy Blood & Tissue Kit, Qiagen). Exons 14 and 15 of the hASS1 gene were amplified using PCR as described above and amplicons were analyzed using 1% agarose gel.

PCR products from ASS1-HLSCs and ASS1-HLSCs treated with HLSC-EVs were analyzed for mutations (c.1087C > T and c.168G > A) using the ABI PRISM SNaPshot Multiplex Kit (Thermo Fisher Scientific), according to the protocol supplied by the manufacturer (normal HLSCs served as control). The SNaPshot method is based on the dideoxy single-base extension of unlabeled oligonucleotide primers. For each of the two mutations analyzed above, a primer annealing adjacent to the potentially mutant nucleotide was developed (Table 2). Briefly, the SNaPshot reaction was performed in a volume of 10 μl, containing 3 μl PCR product, 5 μl ready reaction mix, 1× sequencing buffer, and SNaPshot primers concentrated at 0.02 pmol/μl. At the end of the reaction, the labeled products were first treated with shrimp alkaline phosphatase (SAP; USB Corporation) to remove excess dideoxynucleotide triphosphate and then separated on 36-cm-long capillaries by performing a 25-min run in an automatic sequencer (ABI PRISM 3500 Genetic Analyzer, Thermo Fisher Scientific). GeneScan Analysis Software version 3.7 (Thermo Fisher Scientific) was used for data analysis. For each sample, the intensity of the fluorescent peak was measured.

### Statistical analysis

Results are expressed as mean ± standard deviation (SD). Statistical analysis was performed using the Student’s *t* test and analysis of variance (ANOVA) with Newmann-Keuls test or ANOVA with Dunnet’s multicomparison tests where appropriate. A *p* value <0.05 was considered significant.

## Results

### Characterization and in vitro differentiation of citrullinemia type I-derived ASS1-HLSCs

**Fig. 1:**
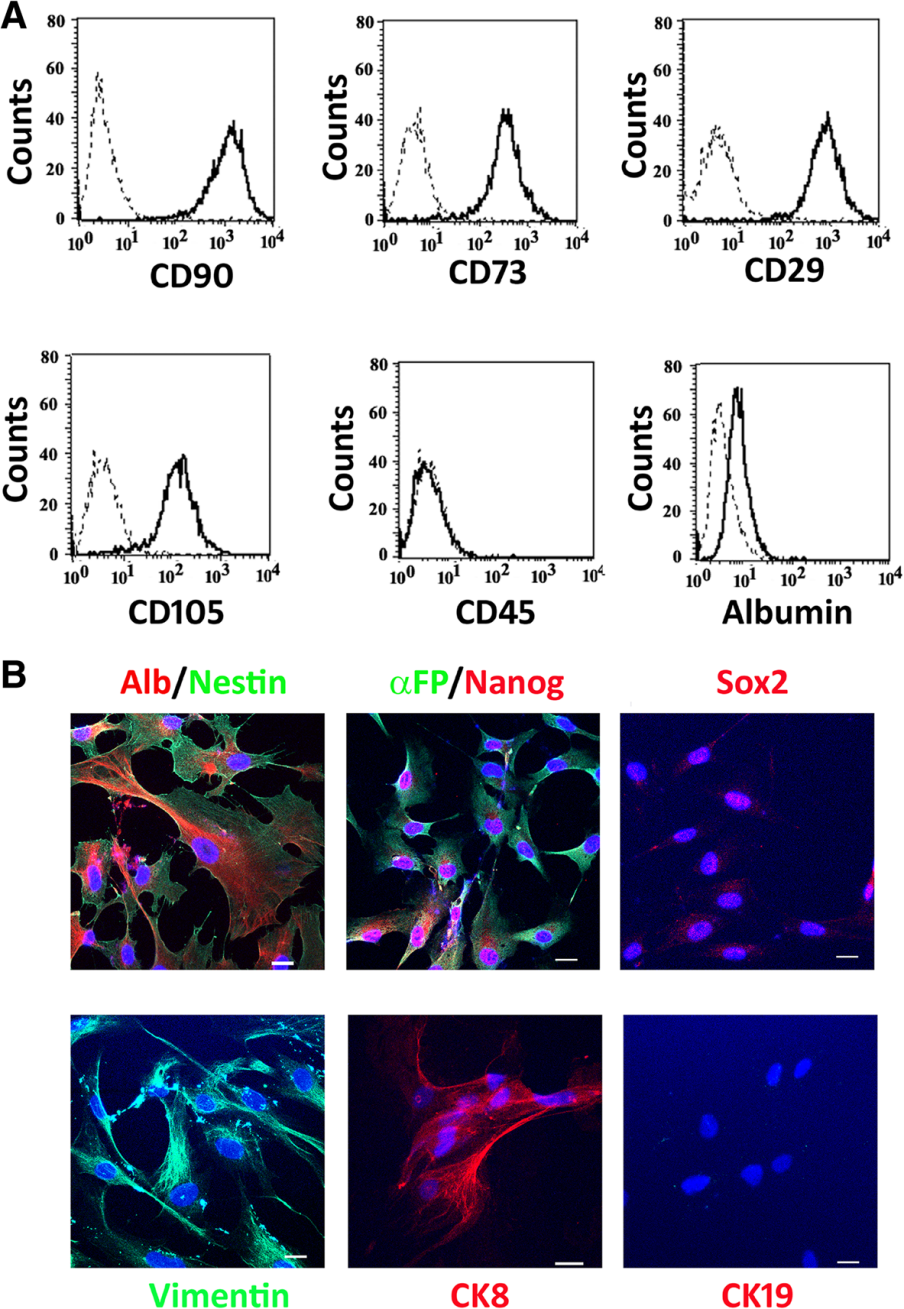
Characterization of ASS1-HLSCs. **a** Representative flow cytometric analysis of markers expressed by ASS1-HLSC (*black histograms*; *dotted histograms* represent the isotypic controls). Cells were labeled with FITC- or PE-conjugated antibody. One hundred percent of ASS1-HLSCs were positive for CD90, CD73, CD29, and CD105, and negative for CD45 as described previously for normal HLSCs [4]. ASS1-HLSCs were also positive for the liver marker albumin [4]. Data represent one of three experiments with similar results. **b** Representative immunofluorescence micrographs of ASS1-HLSCs stained with antibodies against albumin, nestin, α-fetoprotein (α*-FP*), nanog, sox2, vimentin, cytokeratin (*CK*)8 and CK19. Hoechst dye 33258 was applied for nuclei staining. *Scale bars* = 5 μm. Data represent one of three experiments with similar results

ASS1-HLSCs derived from the liver of a patient with citrullinemia type I were characterized to confirm their expression of stem cell markers similar to HLSCs. Flow cytometric analysis revealed that ASS1-HLSCs, like their normal counterparts, were positive for several mesenchymal stem cell markers such as CD90, CD73, CD29, and CD105 (Fig. 1a). Furthermore, they were positive for the liver tissue-specific marker albumin but negative for the hematopoietic marker CD45 (Fig. 1a). In addition, immunofluorescence microscopy confirmed ASS1-HLSCs to be positive for the hepatocyte precursor marker α-FP, as well as vimentin (100%), nestin (100%), and CK8 (40%) (Fig. 1b). In contrast, CK19, a marker for oval cells, was negative (Fig. 1b). To assess the in vitro differentiation capabilities of ASS1-HLSCs, they were induced to differentiate into endothelial, osteogenic, and adipogenic lineages. Similar to HLSCs, ASS1-HLSCs were able to undergo endothelial differentiation when cultured in endothelial induction medium (Additional file 1: Figure S1). After 3 weeks in culture, ASS1-HLSCs compared to undifferentiated cells expressed de novo endothelial markers such as KDR, CD31, VE-cadherin, and von Willebrand Factor (vWF). Furthermore, when cultured in osteogenic differentiation medium, ASS1-HLSCs transformed morphologically from spindle shaped cells to cuboidal cells and underwent mineralization as reflected by alizarin staining (Additional file 1: Figure S1). ASS1-HLSCs when maintained in adipogenic differentiation medium did not differentiate into adipocytes as was also observed with normal HLSCs (data not shown).

**Fig. 2:**
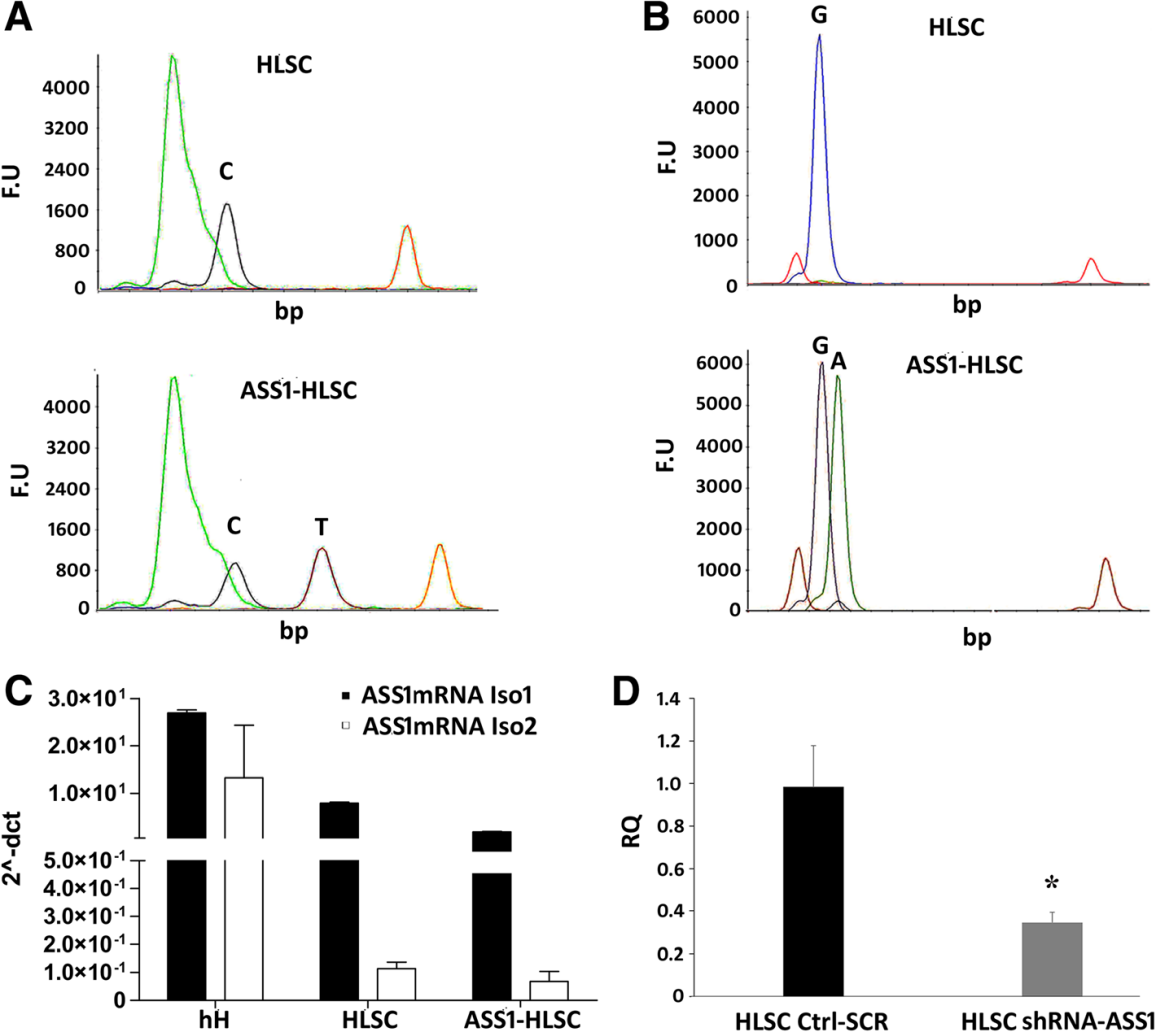
The expression of ASS1 gene and ASS1 mRNA in HLSCs and ASS1-HLSC. **a**,**b** Representative SNaPshot sequences of ASS1 gene in normal human liver stem cells (*HLSCs*) and HLSCs from the liver of a patient suffering from citrullinemia type 1 (*ASS1-HLSCs*). **a** The upper electropherogram shows a normal ASS1 gene profile from HLSCs; the lower electropherogram shows the mutated gene profile from ASS1-HLSCs (c.1087C > T). **b** The upper electropherogram shows a normal ASS1 gene profile; the lower electropherogram shows the mutated ASS1-HLSC gene profile (c.168G > A). Data represent one of three experiments with similar results. **c** qRT-PCR analysis showing the expression of ASS1 isoforms 1 (*Iso1*) and 2 (*Iso2*) mRNA in human hepatocytes (*hH*), HLSCs, and ASS1-HLSCs. hH were used as positive control. **d** qRT-PCR analysis of ASS1 Iso1 mRNA in HLSCs transfected with ASS1 shRNA. Data represent the mean of two independent experiments performed in triplicate. *p < 0.05, HLSC shRNA-ASS1 vs HLSC Ctrl-SCR. *A* adenine, *C* cytosine, *F.U* fluorescence unit, *G* guanine, *SCR* scramble, *T* thymine

In order to identify and characterize the ASS1 mutations in ASS1-HLSCs, a SNaPshot sequencing analysis was implemented. Briefly, after DNA extraction, a first amplification step was performed using two different specific primers for the genomic regions of the ASS1 gene expressing two potential codon mutations (g.55277 C > T, g.59839 G > A). The PCR products were then analyzed for mutations using the ABI PRISM SNaPshot Multiplex Kit. As shown in Fig. 2a and b, the mutations in ASS1-HLSCs were confirmed by the presence of the mutated bases T and A which were absent in normal HLSCs. At variance to mature hH that expressed both isoform 1 and 2 of ASS1 mRNA, the undifferentiated HLSCs and ASS1-HLSCs expressed mainly isoform 1 (Fig. 2c). Figure 2d shows the reduction of ASS1 mRNA expression in HLSCs silenced for the ASS1 gene.

### Expression of ASS1 protein

**Fig. 3:**
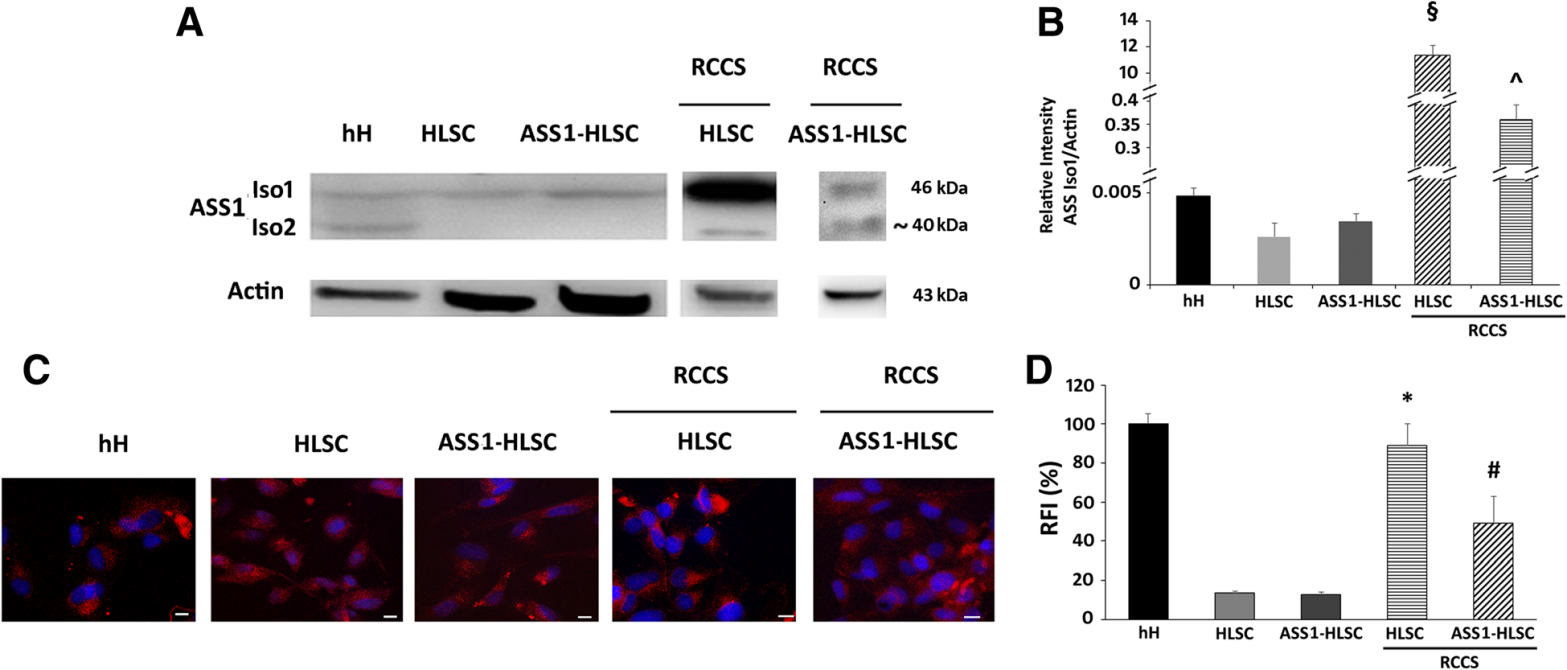
The expression of ASS1 protein in HLSCs and ASS1-HLSCs. **a** Western blot confirming the presence or absence of ASS1 protein isoform 1 (*Iso1*) and isoform 2 (*Iso2*) in 10 μg total protein extracted from human hepatocytes (*hH*), human liver stem cells (*HLSCs*), HLSCs from the liver of a patient suffering from citrullinemia type 1 (*ASS1-HLSCs*), and Rotary Cell Culture System (RCCS)-differentiated HLSCs and ASS1-HLSCs. Actin was run on a parallel gel as a representative of loading. **b** Quantification of the relative band intensity of ASS1 Iso1 across all samples in Fig. 3a normalized to actin. **c** Representative immunofluorescence micrographs of hH, HLSCs, ASS1-HLSCs, and RCCS-differentiated HLSCs and ASS1-HLSCs stained for ASS1 enzyme. The nuclei of the cells were stained with Hoechst dye 33258. Original magnification × 400, *scale bar* = 50 μm. Data represent one of two experiments with similar results. **d** Quantification of the relative fluorescence intensity (*RFI*) of all the cell culture conditions in Fig. 3c with hH serving as a positive control established as the 100%. Data represent the mean of three independent experiments with similar results. ^§^ *p* < 0.05, HLSCs/RCCS vs HLSC; ^^^ *p* < 0.05, ASS1-HLSC/RCCS vs ASS1-HLSC; *p < 0.05, HLSC/RCCS vs HLSC; ^#^ *p* < 0.05, ASS1-HLSC/RCCS vs ASS1-HLSC

The expression of ASS1 protein in mature hH, HLSCs, and ASS1-HLSCs was evaluated by Western blot (Fig. 3a and b). hH expressed two isoforms of ASS1 protein, one at 46 kDa (isoform 1 (Iso1)) and the other at 40 kDa (isoform 2 (Iso2)) (Fig. 3a). Consistent with the low expression of Iso2 mRNA in undifferentiated HLSCs and ASS1-HLSCs, Western blot analysis showed only Iso1. This suggests that under undifferentiating conditions the low amount of Iso2 mRNA was not translated into protein. However, differentiation into hepatocytes via RCCS induced the expression of the Iso2 for both HLSCs and ASS1-HLSCs (Fig. 3a). Moreover, after differentiation the expression of Iso1 was increased in both HLSCs and ASS1-HLSCs compared to the undifferentiated counterparts (Fig. 3a and b).

The expression of the ASS1 protein in hH, HLSCs, and ASS1-HLSCs was further confirmed by immunofluorescence staining (Fig. 3c). As seen in Fig. 3d, quantitative analysis of ASS1 protein expression shows a significant increase after RCCS-induced differentiation (Fig. 3c and d). These data therefore confirm that differentiation to hepatocytes of both the wild-type and mutated HLSCs significantly increases the expression of the ASS1 protein.

### Characterization of HLSC-EVs

**Fig. 4:**
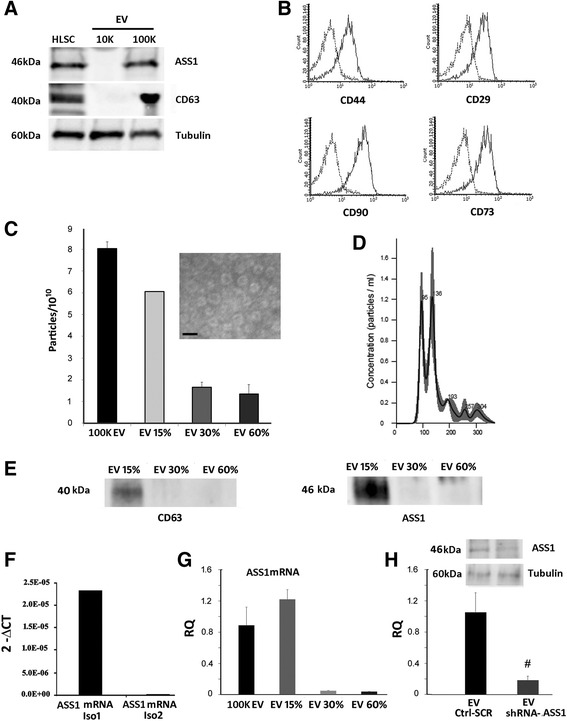
Characterization of HLSC-derived EVs. **a** Representative Western blot analysis of ASS1 and CD63 expression in human liver stem cells (*HLSCs*) and the 10 K and 100 K fractions of HLSC-derived extracellular vesicles (*EVs*) obtained by differential ultracentrifugation. Tubulin was run on a parallel gel as a representative of loading (10 μg protein). Data represent one of two experiments with similar results. **b** Flow cytometric analysis of mesenchymal stem cell markers expressed by the 100 K fraction of HLSC-EVs. The 10 K fraction also expresses mesenchymal stem cell markers (data not shown). Data represent one of two experiments with similar results. **c** NanoSight quantification of HLSC-EVs obtained from the 100 K fraction before and after floating separation on iodixanol gradients (data represent a mean of three different experiments). *Insert*: representative electron microscopy of 100 K fraction of HLSC-EVs (*scale bar* = 100 nm). **d** NanoSight size distribution graph showing the quantity and size of HLSC-EVs obtained from the 15% iodixanol gradient. **e** Representative Western blot analysis of CD63 and ASS1 protein expression in HLSC-EVs isolated from different iodixanol fractions. Data represent one of two experiments with similar results. **f** Expression levels of ASS1 isofrom 1 (*Iso1*) and isoform 2 (*Iso2*) mRNA in 100 K HLSC-EVs. **g** Expression levels of ASS1 Iso1 mRNA in EVs isolated from different iodixanol fractions. **h** ASS1 mRNA Iso1 expression in EVs derived from ASS1 shRNA transfected HLSCs compared with EVs derived from HLSCs transfected with the scramble (*SCR*). *Insert*: representative Western blot analysis of ASS1 protein expression in EVs isolated from shRNA transfected HLSCs (*EV shRNA-ASS1*) and from EVs isolated from HLSCs transfected with the scramble shRNA (*EV Ctrl-SC*R). Data represent the mean of three independent experiments performed in triplicate. ^#^ *p* < 0.05, EV shRNA-ASS1 vs EV Ctrl-ASS1

On analyzing the 10 K and 100 K fractions, ASS1 protein expression was observed only in the 100 K fraction concurrently with the expression of the exosomal marker CD63 (Fig. 4a). Furthermore, the same fraction was also positive for the mesenchymal markers typical of HLSCs as observed by fluorescence-activated cell sorting (FACS) analysis (Fig. 4b). No further analysis was performed on the 10 K fraction as it was negative for ASS1 and CD63 (Fig. 4a).

The 100 K fraction was also submitted to floating separation whereby 75% of the EVs were detected in the 15% iodixanol fraction with a mean size of 155 ± 3.6 nm as determined by NanoSight analysis (Fig. 4c and d). In addition, transmission electron microscopy revealed a homogenous layer of these vesicles with a morphology resembling exosomes with a smaller size than observed by NanoSight (Fig. 4c, insert). Furthermore, on analyzing the EVs obtained from the three iodixanol fractions by Western blot, only the 15% fraction was positive for the exosomal marker CD63 as well as the ASS1 protein (Fig. 4e).

PCR analysis also confirmed at a molecular level the presence of mRNA for ASS1 Iso1 but not for ASS1 Iso2 in 100 K HLSC-EVs (Fig. 4f) and in the 15% iodixanol EV fraction compared to the other fractions (Fig. 4g).

In order to evaluate whether restoration of ASS1 enzymatic activity was observed in ASS1-HLSCs treated with HLSC-EVs, we generated EVs depleted of the ASS1 enzyme by silencing the gene at a cellular level. As shown in Fig. 4h, EVs derived from HLSC shRNA-ASS1 were depleted of both Iso1 ASS1 mRNA and protein.

### EVs derived from normal HLSCs increased urea production and ASS1 enzymatic activity in mutated ASS1-HLSCs

**Fig. 5:**
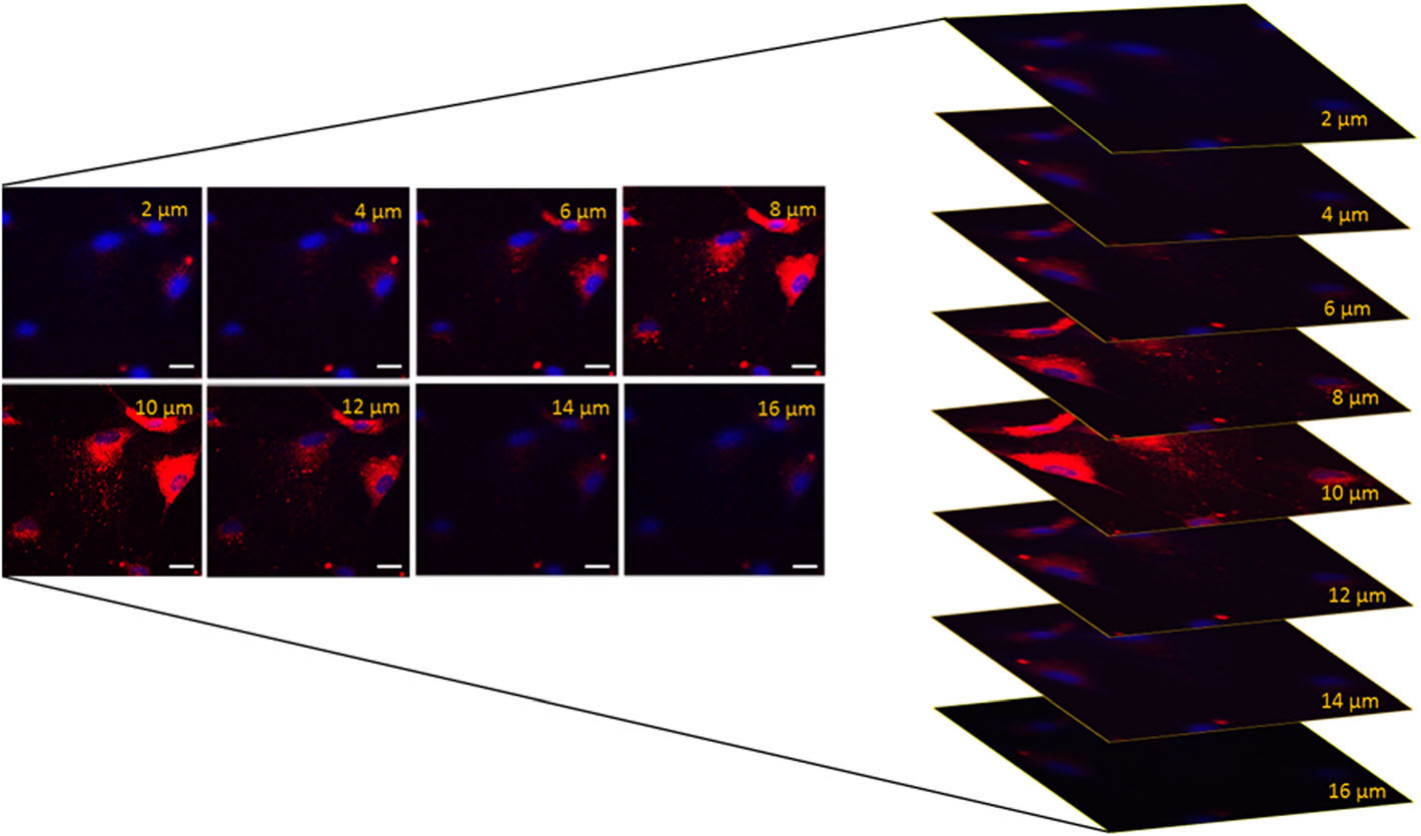
Internalization of HLSC-EVs in hepatocytes differentiated from ASS1-HLSCs. Representative confocal microscopy showing the internalization of 1 × 10^10^ Dil-labeled EVs in hepatocytes differentiated from ASS1-HLSCs after 6 h of incubation at 37 °C. *Z* stack analysis shows the presence of EVs within the cytoplasm of the cells indicating an effective uptake of vesicles; *scale bar* = 50 μm. Data represent one of three experiments performed with similar results

**Fig. 6:**
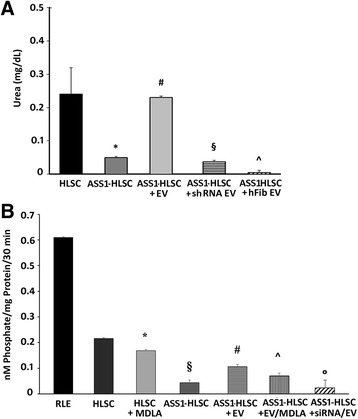
Urea production and ASS1 enzymatic activity. **a** Histogram representing the urea production ability of hepatocytes differentiated under RCCS conditions for 4 days from human liver stem cells (*HLSCs*), HLSCs from the liver of a patient suffering from citrullinemia type 1 (*ASS1-HLSCs*), ASS1-HLSCs treated with 1 × 10^10^ HLSC-derived extracellular vesicles (*EVs*), ASS1-HLSCs treated with 1 × 10^10^ HLSC-EVs from ASS1 shRNA transfected HLSCs (*shRNA EVs*) or with 1 × 10^10^ EVs derived from human fibroblasts (*hFib EV*). Data are expressed as mean ± SD of four different experiments. ANOVA with Newmann-Keuls multicomparison tests was performed. *p < 0.05, ASS1-HLSC vs HLSC; ^#^ *p* < 0.05, ASS1-HLSC + HLSC-EVs vs ASS1-HLSC; ^§^ *p* < 0.05, ASS1-HLSC + shRNA EVs vs ASS1-HLSC + HLSC-EVs; ^^^ *p* < 0.05 ASS1-HLSC + hFib EVs vs AS-HLSC + EVs. **b** The enzymatic activity of ASS1 immunoprecipitated from either rat liver extract (*RLE*; positive control), hepatocytes differentiated in RCCS for 4 days from HLSCs, ASS1-HLSCs, or ASS1-HLSCs treated with 1 × 10^10^ HLSC-EVs were measured. For selected experiments the enzyme activity was measured in the presence of α-methyl-dl-aspartic acid (*MDLA*), a specific inhibitor of ASS1 enzymatic activity, and using ASS1-HLSCs previously transfected with ASS-siRNA. Data are expressed as mean ± SD of four separate experiments. ANOVA with Newmann-Keuls multicomparison tests was performed. *p < 0.05, HLSC + MDLA vs HLSC; ^§^ *p* < 0.05, ASS1-HLSC vs HLSC; ^#^ *p* < 0.05, ASS1- HLSC + EVs vs ASS1-HLSC; ^^^ *p* < 0.05, ASS1- HLSC + EV/MDLA vs ASS1-HLSC + EV; °*p* < 0.05, ASS1-HLSC + siRNA/EV vs ASS1-HLSC + EV

As shown in Fig. 5, HLSC-EVs were internalized in ASS1-HLSCs after 6 h of incubation at 37 °C. It has been described that, as a consequence of the genetic mutations, the ASS1 enzyme activity is decreased in fibroblasts, liver, and in all ASS1-expressing tissues [2]. In order to evaluate whether HLSC-EVs were able to correct the defective urea production and enzymatic activity in ASS1-HLSCs, ASS1-HLSCs were treated with HLSC-EVs (1 × 10^10^ particles) in RCCS for 4 days. At the end of the experiments, urea production and ASS enzymatic activity were measured (Fig. 6a and b). Mutated ASS1-HLSCs produced significantly less urea and showed a decreased ASS1 enzymatic activity than normal HLSCs. HLSC-EV treatment significantly increased urea production and enzymatic activity in ASS1-HLSCs. The specificity of the enzymatic activity reaction was confirmed by the inhibitory effect of treating the cells with MDLA as a specific inhibitor of the enzyme (Fig. 6b). ASS1 enzymatic activity was also inhibited in HLSC-EV-treated ASS1-HLSCs when the cells were transfected with ASS1-siRNA (Fig. 6b). ASS1 shRNA-depleted HLSC-EVs were unable to restore urea production in ASS1-HLSCs (Fig. 6a). The ability of HLSC-EVs to restore urea production was specific since EVs obtained from human fibroblasts (hFib) which did not contain ASS1 protein and only a low level of ASS1 mRNA (data not shown) were ineffective (Fig. 6a). Moreover, the reduction of ASS1 urea production by EVs derived from hFib also depended on an inhibition of cell differentiation in RCCS (data not shown).

### HLSC-EV treatment decreased DNA mutations in ASS1-HLSCs

**Fig. 7:**
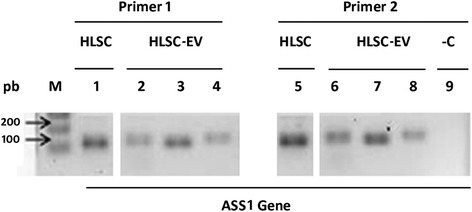
The expression of the ASS1 gene in HLSCs and EVs. ASS1 gene expression was determined in human liver stem cell-derived extracellular vesicles (*HLSC-EVs*) by PCR analysis. Two different regions of human ASS1 (CTLN1) gene located in exons 14 and 15 were amplified by PCR with two different primers that amplified both the regions respectively; exon 14 (*Primer 1*), exon 15 (*Primer 2*). A PCR without the presence of nucleotides was used as negative control (*–C*). Three different preparations of EVs (lanes 2, 3, and 4 and lanes 6, 7, and 8) were analyzed in comparison with the cells of origin (HLSCs; lanes 1 and 5)

**Fig. 8:**
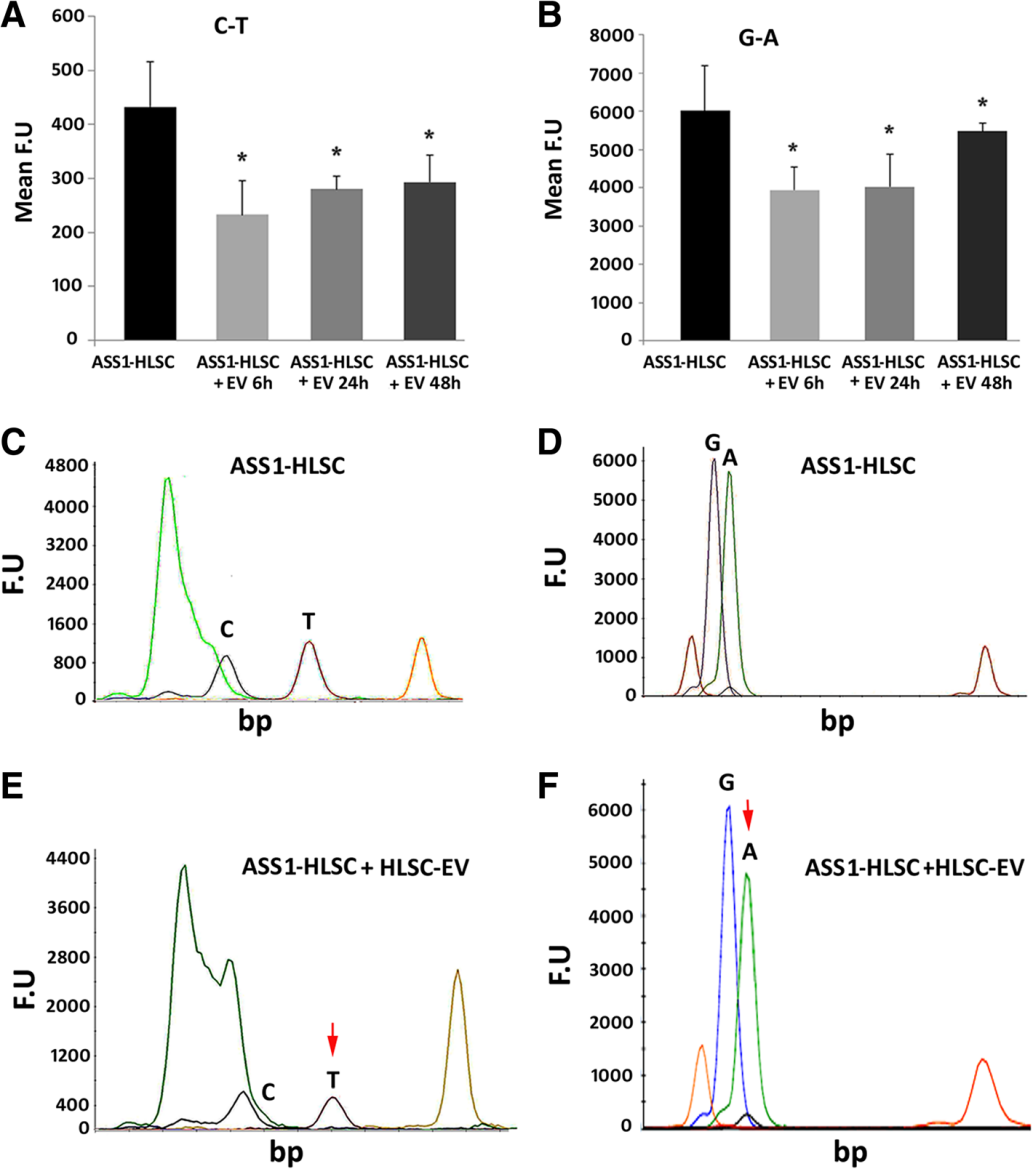
ASS1 mutations. **a**,**b** SNaPshot method was used to characterize the mutations in human liver stem cells (*HLSCs*) from the liver of a patient suffering from citrullinemia type 1 (*ASS1-HLSCs*). ASS1-HLSCs were treated with 1 × 10^10^ HLSC-derived extracellular vesicles (*EVs*) in adherence culture conditions for 6, 24, and 48 h. At the end of the experiments, DNA was recovered from ASS1-HLSCs treated with or without EVs and sequencing mutation experiments were performed to detect C-T mutations (**a**) and G-A mutations (**b**). Data are expressed as mean ± SD of three individual experiments. ANOVA with Dunet’s multicomparison tests was performed. *p < 0.05, all experimental conditions vs ASS1-HLSC. **c**–**d** Representative electropherograms of ASS1-HLSCs performed to detect C-T mutation (**c**) and G-A mutation (**d**). **e-f** Representative electropherograms of ASS1-HLSCs treated for 6 h with 1 × 10^10^ HLSC-EVs showing the reduction of C-T mutation (**e**) and G-A mutation (**f**). *A* adenine, *C* cytosine, *F.U* fluorescence unit, *G* guanine, *T* thymine

Since HLSC-EVs carry fragments of ASS1 DNA (Fig. 7), we evaluated whether they may affect the mutations when incorporated into ASS1-HLSCs. We performed SNaPshot sequencing to determine the contribution of HLSC-EVs to correction of both mutations present in ASS1-HLSCs. Figure 8 shows that the amplitude of picks of the mutated bases T and A belonging to one allele of the ASS1 region of ASS1-HLSCs was significantly reduced when cells were cultured in adhesion culture for 6, 24, and 48 h with HLSC-EVs (1 × 10^10^ particles).

## Discussion

In the present study we demonstrated that EVs derived from normal HLSCs increased the production of urea and ASS1 enzymatic activity in hepatocytes differentiated from HLSCs derived from a patient with Citrullinemia type I.

Citrullinemia type I is an inherited disorder of the urea cycle caused by a genetic mutation in the ASS1 gene. The absence of a functional form of ASS1 enzyme leads to a rapid accumulation of ammonia and other toxic substances in the blood leading to an early neonatal death. Liver transplantation remains the only curative treatment for this defect. However, due to limited availability of donor livers, substitute therapies are currently being researched [23].

Recently, Syres et al. demonstrated that the accumulation of cysteine in organs was decreased after the injection of mesenchymal stem cells in a cysteinotic knockout mice model (CTNA^–/–^) [24]. The cells were found in multiple organs resulting in a 90% decrease in tissue cysteine levels, leading to the recovery of kidney injury [24]. Nevertheless, the reduction in tissue cysteine levels could not be attributed to mesenchymal stem cell transdifferentiation or fusion with resident CTNA mutated cells. In further studies, to explain the mechanism of reduction of intralysosomal accumulation of cysteine, Iglesias et al. used an in vitro coculture model of CTNA^–/–^ fibroblasts and human amniotic mesenchymal stem cells [25]. They demonstrated that the uptake of mesenchymal stem cell-derived EVs carrying wild-type cysteinosin protein and mRNA by mutant fibroblasts significantly reduced the lysosomal accumulation of cysteine in the cells. Katsuda et al. recently showed that EVs derived from adipose mesenchymal stem cells may transfer a functional enzyme to target cells [26]. In addition, several studies provided evidence of effective transfer of functional mRNA, miRNA, and DNA by EVs [27–29].

Collectively, these works suggest that EVs could interact with target cells and transfer functional protein and nucleic acids that may modify their phenotype. In agreement with this evidence, we found that HLSC-EVs contained wild-type ASS1 protein, mRNA, and DNA. Furthermore, on incubating these EVs with mutated ASS1-HLSCs the mutated enzymatic effect was corrected, restoring urea production and enzymatic activity. Undifferentiated HLSCs expressed Iso1 and, to a significantly less extent, Iso2 mRNA, and the amount of Iso2 protein was undetectable by Western blot analysis. Consistently, EVs secreted from undifferentiated HLSCs contained only mRNA and protein of Iso1. In addition, knocking down Iso1 by shRNA in HLSCs depleted the levels of ASS1 mRNA and protein in the EVs released subsequently by the cells. These EVs from ASS1 shRNA knockdown HLSCs were unable to restore urea production in hepatocytes differentiated from ASS1-HLSCs. This result suggests that the biological activity of EVs may depend on the transfer of both functional ASS1 protein and mRNA. It is unclear from this experiment to what extent protein/mRNA transfer contributed to the recovery of ASS1 enzymatic activity in ASS1-HLSCs. However, the result of the experiment using transfection of ASS1-HLSCs with ASS1-siRNA that shows inhibition of HLSC-EV-induced correction of enzymatic activity suggests a predominant role of mRNA transfer.

In addition, we also found that HLSC-EVs carried fragments of the nonmutated ASS1 gene and that the amplitude of the peak of the bases mutated was reduced in ASS1-HLSCs after treatment with vesicles. However, the transfer of DNA was not relevant for the correction of the enzyme activity in ASS1-HLSCs as EVs derived from HLSCs silenced with ASS1 shRNA (which only diminished ASS1 mRNA levels) had a significant reduction in their biological activity.

Further studies are required to evaluate whether the horizontal transfer of DNA could also correct the enzymatic defect in the mutated cells. Nevertheless, several studies have recently investigated the possibility of horizontal transfer of DNA by EVs. For instance, Lázaro-Ibáñez et al. [30] reported the presence of double-stranded genomic DNA fragments in exosomes derived from prostate cancer cells and suggested the transfer of oncogenes between cancer and noncancerous cells. Fischer et al. demonstrated the horizontal transfer of DNA genes by EVs in recipient cells [31]. These studies suggest that vesicles containing genomic DNA may be instrumental in the exchange of genetic information between eukaryotic cells and open the possibility to exploit this mechanism for correction of genetic disorders.

The mechanisms of exosomal biogenesis and sorting of proteins and nucleic acids are only partially known. However, recently the mechanisms controlling specific miRNA sorting in hepatocyte exosomes has been described by Santangelo et al. [32]. They identified an RNA binding protein SYNCRIP as a critical component of miRNA accumulation within exosomes, suggesting a possible selective modulation of exosome cargo. EVs are enriched in ribonucleoproteins that may be involved in the storage, traffic, and protection of degradation of ribonucleic acid [33]. Moreover, the association of Alix, a component of endosomal sorting complex required for transport, with Ago2, which is involved in miRNA maturation, has been shown to contribute to miRNA accumulation into EVs [34].

## Conclusions

EVs derived from stem cells may act as paracrine mediators by transferring proteins and nucleic acids inducing epigenetic changes in recipient cells. Herein, we found that EVs derived from normal HLSCs have the potential to correct ASS1 enzyme deficiency in hepatocytes differentiated from HLSCs derived from a patient with citrullinemia type I, suggesting a potential application of EVs derived from stem cells in some inherited diseases.

## Additional file

Additional file 1: Figure S1. In vitro differentiation of ASS1-HLSCs. (A) Representative flow cytometric analysis of undifferentiated ASS1-HLSCs showing negative expression of KDR, CD31, and VE-Cadherin. Von Willebrand factor (vWF) expression and alizarin red staining for mineralization was also negative in undifferentiated conditions as observed by microscopy. (B) Representative flow cytometric analysis of ASS1-HLSCs after endothelial differentiation showing the presence of KDR, CD31, and VE-Cadherin (black histograms; dotted histograms represent isotypic controls). Representative micrographs showing the expression of vWF in ASS1-HLSCs after endothelial differentiation, and osteogenic differentiation of ASS1-HLSCs showing positive staining for calcium deposits as indicated by alizarin 21 days after culturing in osteogenic differentiation medium. Scale bar = 50 μm. Data represent one of three experiments performed with similar results. (TIF 8299 kb)

## Abbreviations

ASS: Argininosuccinate synthase
ASS1-HLSC: Human liver stem cell from the liver of a patient suffering from citrullinemia type I
CK: Cytokeratin
DMSO: Dimethyl sulfoxide
EV: Extracellular vesicle
FACS: Fluorescence-activated cell sorting
FITC: Fluorescein isothiocyanate
hFib: human fibroblasts
hH: Human hepatocytes
HLSC: Human liver stem cell
Iso: Isoform
MDLA: α-Methyl-dl-aspartic acid
PBS: Phosphate-buffered saline
PE: Phycoerythrin
Pi: Inorganic phosphate
qRT-PCR: Quantitative real-time polymerase chain reaction
RCCS: Rotary Cell Culture System
RPMI: Roswell Park Memorial Institute
αFP: α-Fetoprotein
