# Supplementary figures and images for: Extracellular vesicles from human liver stem cells restore argininosuccinate synthase deficiency
Source: Stem Cell Res Ther. 2017 Jul 27;8:176. doi: 10.1186/s13287-017-0628-9 (PMC5531104; doi:10.1186/s13287-017-0628-9)

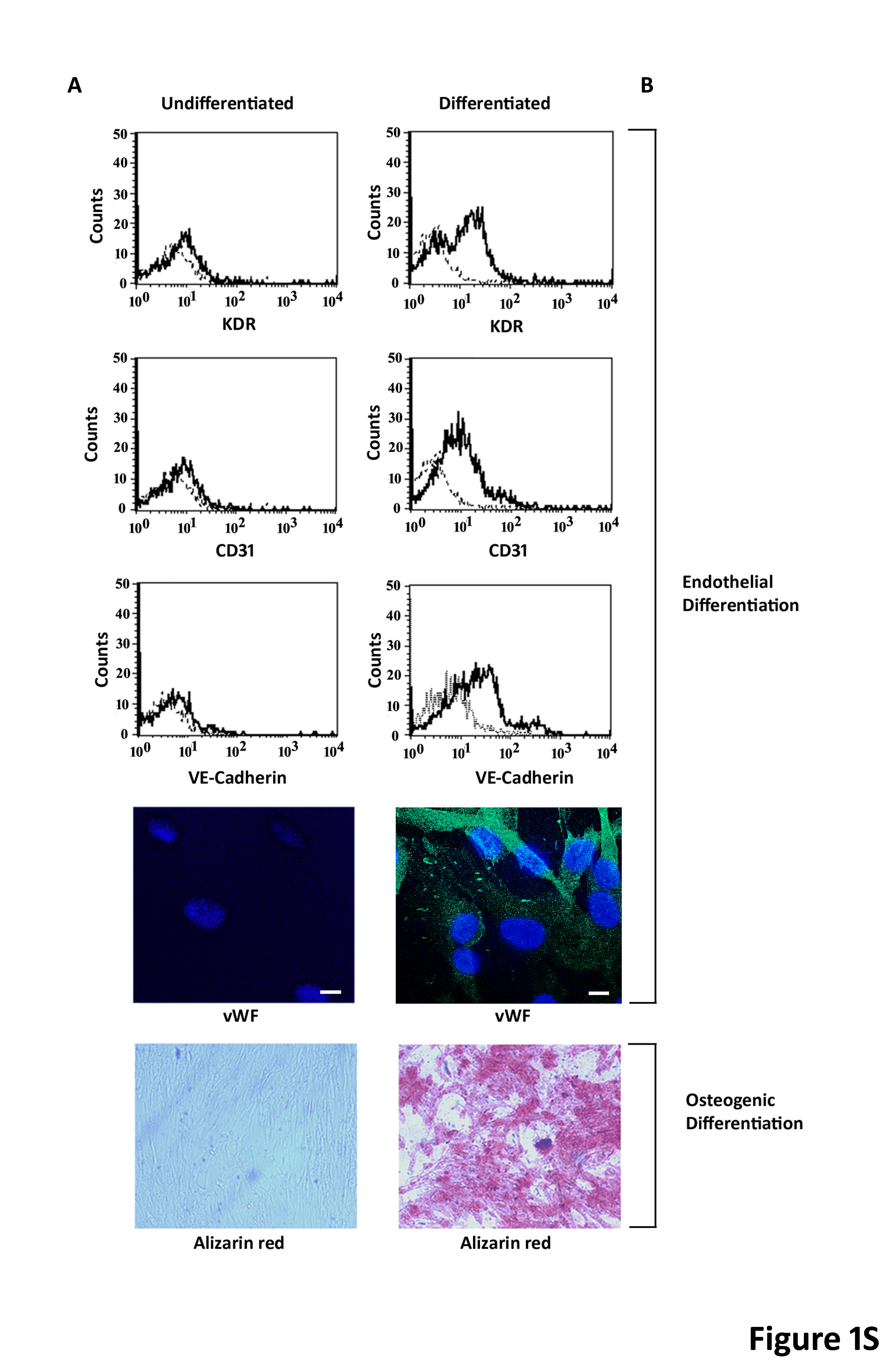

Supplement: Additional file 1: Figure S1. — In vitro differentiation of ASS1-HLSCs. (A) Representative flow cytometric analysis of undifferentiated ASS1-HLSCs showing negative expression of KDR, CD31, and VE-Cadherin. Von Willebrand factor (vWF) expression and alizarin red staining for mineralization was also negative in undifferentiated conditions as observed by microscopy. (B) Representative flow cytometric analysis of ASS1-HLSCs after endothelial differentiation showing the presence of KDR, CD31, and VE-Cadherin (black histograms; dotted histograms represent isotypic controls). Representative micrographs showing the expression of vWF in ASS1-HLSCs after endothelial differentiation, and osteogenic differentiation of ASS1-HLSCs showing positive staining for calcium deposits as indicated by alizarin 21 days after culturing in osteogenic differentiation medium. Scale bar = 50 μm. Data represent one of three experiments performed with similar results. (TIF 8299 kb) [file 13287_2017_628_MOESM1_ESM.tif]
